# A Thermal Anemometry Method for Studying the Unsteady Gas Dynamics of Pipe Flows: Development, Modernisation, and Application

**DOI:** 10.3390/s23249750

**Published:** 2023-12-11

**Authors:** Leonid Plotnikov

**Affiliations:** Department of Turbines and Engines, Ural Federal University Named after the First President of Russia B.N. Yeltsin, Str. Mira 19, 620002 Yekaterinburg, Russia; leonplot@mail.ru; Tel.: +7-922-291-64-50

**Keywords:** constant-temperature hot-wire anemometer, electronic circuit, protection unit, stationary and pulsating gas flows, comparative analysis, technical characteristics and verification

## Abstract

A detailed study of the gas-dynamic behaviour of both liquid and gas flows is urgently required for a variety of technical and process design applications. This article provides an overview of the application and an improvement to thermal anemometry methods and tools. The principle and advantages of a hot-wire anemometer operating according to the constant-temperature method are described. An original electronic circuit for a constant-temperature hot-wire anemometer with a filament protection unit is proposed for measuring the instantaneous velocity values of both stationary and pulsating gas flows in pipelines. The filament protection unit increases the measuring system’s reliability. The designs of the hot-wire anemometer and filament sensor are described. Based on development tests, the correct functioning of the measuring system was confirmed, and the main technical specifications (the time constant and calibration curve) were determined. A measuring system for determining instantaneous gas flow velocity values with a time constant from 0.5 to 3.0 ms and a relative uncertainty of 5.1% is proposed. Based on pilot studies of stationary and pulsating gas flows in different gas-dynamic systems (a straight pipeline, a curved channel, a system with a poppet valve or a damper, and the external influence on the flow), the applications of the hot-wire anemometer and sensor are identified.

## 1. Introduction

Thermal anemometry is widely and actively used in all areas of science, engineering, and technology due to its versatility, accuracy, and efficiency [1,2]. Generally, a measuring system based on this method contains a hot-wire anemometer (HWA) and a thermal sensor and is designed to study the gas-dynamic and heat transfer characteristics of liquid and gas flows. Today, there is a wide variety of HWA sensor designs and several hot-wire anemometer operating principles for specific research objectives. Scientists and specialists continue to develop measurement systems based on hot-wire anemometry in order to increase accuracy, reliability, and versatility.

The following is a brief overview of the latest developments in improving the operation of hot-wire anemometers and the design of HWA sensors. R. A. Gomes and R. Niehuis proposed an original hot-wire anemometer design without a Wheatstone measuring bridge [3]. In this case, the temperature of the sensing element (filament) or current was digitally controlled with an FPGA unit. A comparison of the original and traditional HWA systems showed the identity of the data on measuring laminar and turbulent gas flows. Y. Tang et al. proposed a way to increase the sensitivity of constant-temperature hot-wire anemometers using cladding-etched optical fibre Bragg grating [4]. Based on experimental research, the authors showed that the measuring system’s sensitivity increased by up to 30% compared to traditional HWAs and sensors. M. Schniedenharn and colleagues developed an automated mechatronic system that accurately places a hot-wire anemometer sensor inside an experimental bench [5]. The use of HWAs along with a mechatronic system for installing a sensing element made it possible to significantly increase the accuracy of measuring flow velocity and to obtain data on turbulence with spatial resolution. V. P. Khodunkov proposed a method for expanding the dynamic ranges of hot-wire anemometers to study gas flows moving at high speeds [6]. This method’s main idea was to use two HWAs with significantly different thermal inertia to simultaneously measure the flow velocity and heat transfer coefficient. This method’s accuracy is 1.5% according to the author’s experimental estimates.

Scientists and experts have also emphasised how the design of hot-wire anemometer sensors can improve measurement accuracy and advance their functionality. For instance, A. Hewes and L. Mydlarski proposed several design options for sensors measuring the gas-dynamic characteristics of various gases, such as a two-component HWA sensor for the simultaneous measurement of helium velocity and concentration in laminar and turbulent flows [7]. They also developed a three-component HWA sensor to simultaneously measure the velocity, helium concentration, and temperature in laminar and turbulent flows [8]. A similar sensor design was developed by L. Pantoli [9], making it possible to simultaneously determine wind speed and direction when studying gas-dynamic effects in the atmosphere. The described sensor designs make it possible to speed up measurements and obtain more accurate and reliable data on the measured physical processes. F. Daniel and colleagues proposed and tested a method for creating various HWA sensor designs with 3D printing [10]. This can create unique sensor configurations designed for specific studies. M.-T. Atienza developed a design version of a film sensor for a constant-temperature hot-wire anemometer to measure the intensity of heat transfer intended for measurements in the atmosphere of Mars [11].

An actively developing area in enhancing measuring systems based on HWAs is the use of optical fibre and the effect of fibre Bragg grating [12,13]. Optical fibre makes it possible to significantly increase the sensitivity of measuring systems, capturing even the slightest fluctuations in gas flow. M. Sekine and M. Furuya compared the technical specifications of the traditional method based on a hot-wire anemometer and HWAs with a fibre-optic sensor [14]. The comparison was made for airflow velocities up to 7 m/s. The results showed identical data for both methods and the higher sensitivity of fibre-optic sensors; the measurement error did not exceed 10%.

A. Pique et al. used another type of HWA sensor for research into turbomachines, namely a nanoscale probe [15]. The need for such sensors is due to the geometric (design) restrictions on the use of large sensing elements. The authors obtained detailed information about the gas-dynamic characteristics of the tip vortex and related phenomena. At the same time, H. Sadeghi and colleagues compared experimental data on the gas dynamics of flows obtained with conventional HWA sensors (with a filament length of up to 1 mm) and a nanosized sensor [16]. Good agreement between the results for both sensors was established. A detailed review of various designs of thermal sensors for hot-wire anemometers and their areas of application is presented in [17].

There are various methods for determining the flow rate of liquid or gas through thermal anemometry [18,19]. For instance, R. E. Bernhardsgrütter et al. proposed a method based on HWAs and a sensor, which allows for the determination of flow characteristics with an accuracy of up to 9%, regardless of the type of liquid [18]. M. Arlit et al. proposed a new method based on a constant-temperature hot-wire anemometer; this made it possible to accurately measure the flow for axially asymmetric velocity profiles after bends, T-joints, or other deformations in pipe geometry [19].

Specialists have paid great attention to the calibration and adjustment of measuring systems based on HWAs and thermal sensors since the accuracy and reliability of research depend on this. Data describing the importance of measuring system calibration are presented in [20,21].

Thus, we can conclude that improving the electronic circuits of hot-wire anemometers and the designs of sensors remains a relevant objective in the development of thermal anemometry for studying the gas-dynamic characteristics of liquid and gas flows.

In order to emphasise the importance of further modernising measuring systems based on HWAs and thermal sensors, it is necessary to briefly describe their applications. For example, thermal anemometry is actively used to study aerodynamics in the aviation industry when examining unsteady flows on aircraft wings [22] or turbulent structures near quadcopter propellers [23]. Thermal anemometry is also widely used in power engineering to study the gas dynamics of pulsating exhaust gas flows in piston engines [24] and flow-through turbomachines [25]. The automotive industry often requires HWAs and various thermal sensors to study the cooling efficiency of ventilated brake discs [26] or to assess the noise level in motor vehicle deflectors [27]. Thermal anemometry is useful for solving problems in the nuclear industry when studying the convective boiling of liquids [28] or when obtaining data on gas dynamics in the vertical core channels of prismatic reactors [29]. HWAs and various sensor configurations are also used in the design of the ventilation ducts of residential buildings [30], the development of electric motors [31], the study of the hydrodynamics of offshore pipelines [32], and so on. Equally, thermal anemometry is actively used to solve classical as well as fundamental scientific problems, such as heated impact jets [33] and the structure of the aerodynamic wake after impacting objects with different geometries [34,35].

Thus, thermal anemometry based on hot-wire anemometers and filament sensors is a relevant tool for obtaining reliable data on the gas dynamics of liquid and gas flows in various technical and scientific applications. Accordingly, the development of this method (the development of original electronic circuits, the creation of new sensor designs, and so on) to increase the accuracy and reliability of experimental data remains urgent.

The key objectives of this study can be formulated as follows:

- To propose an original electronic circuit for a hot-wire anemometer with the function of protecting the sensor’s sensitive element from overheating during the pre-operation setup;
- To develop the design of a hot-wire anemometer and sensor for measuring the instantaneous values of gas flow velocity;
- To confirm the performance of the developed hot-wire anemometer and sensor, as well as evaluate the technical specifications of the measuring system (the time constant and calibration curve);
- To perform experimental studies of stationary and pulsating gas flows in pipelines with different sources of gas-dynamic unsteadiness (a poppet valve, a damper, and compressor blades) in order to assess the correct functioning of the measuring system.

## 2. An Electronic Circuit for a Constant-Temperature Hot-Wire Anemometer with a Filament Overheating Protection Unit

As shown above, a large number of methods, instruments, and equipment have been developed and used in experimental studies on the flow of liquids and gases; therefore, there is now a wide choice. However, not all methods and instruments can be used to measure turbulent non-stationary (pulsating) gas flows. This is due to the fact that the structure of turbulence is complex, chaotic, and three-dimensional. An external influence on the flow can additionally cause pulsations (gas-dynamic unsteadiness) with a wide range of frequencies and amplitudes. These pulsations can be a consequence of the operation of a poppet valve (for example, piston machines), the rotation of a damper (for example, ventilation and air conditioning systems), or a mechanical effect on the flow (for example, turbine and compressor blades). Therefore, a constant-temperature hot-wire anemometer is usually used [1,2]. This choice is due to the fact that hot-wire anemometers have low inertia and high sensitivity, accuracy, and compactness. In this case, the sensitive element of the hot-wire anemometer sensor is a thin metal filament that does not introduce any noticeable distortions during the flow.

**Figure 1:**
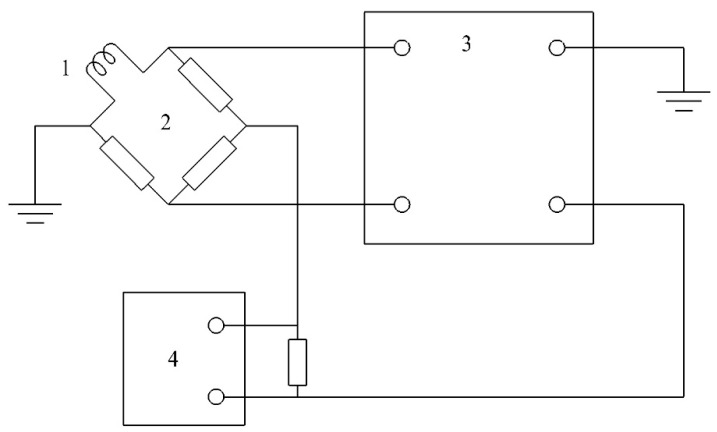
Layout diagram of the electronic unit of a hot-wire anemometer operating according to the constant-temperature method [1]: 1—sensitive element (filament) of the hot-wire anemometer sensor; 2—measuring Wheatstone bridge; 3—differential amplifier; and 4—analogue-to-digital converter (ADC).

A layout diagram of a hot-wire anemometer operating according to the constant-temperature method is shown in Figure 1.

The principle of operation is as follows: The sensitive element (filament) is one of the arms of a resistance bridge, to the measuring diagonal of which a differential amplifier (voltage amplifier and current amplifier) is connected. The output of this amplifier powers the diagonal of the resistance bridge. The electric current passes through the sensing element, thereby heating its metal filament to a certain temperature. The temperature of the sensing element (the filament) is maintained at a constant with a servo-controlled system. The instantaneous amount of electrical energy consumed is equal to the instantaneous heat loss for heating the environment. Heat losses depend on the temperature, pressure, and speed of the medium being measured (a liquid or a gas), as well as the sensing element used (the filament material). If the temperature and pressure of the medium do not change during measurement, then the sensitive element’s current will depend only on the flow speed. If, as a result of an increase in flow speed, the sensing element begins to cool, then its resistance begins to change. A change in resistance leads to a voltage drop in the diagonal of the bridge, which is supplied to the input of the amplifier. This voltage is amplified and fed back to the bridge so that the amplifier current, which is used to heat the filament, increases and compensates for its cooling. Thus, the voltage characterising the heating of the filament is a measure of the flow rate.

**Figure 2:**
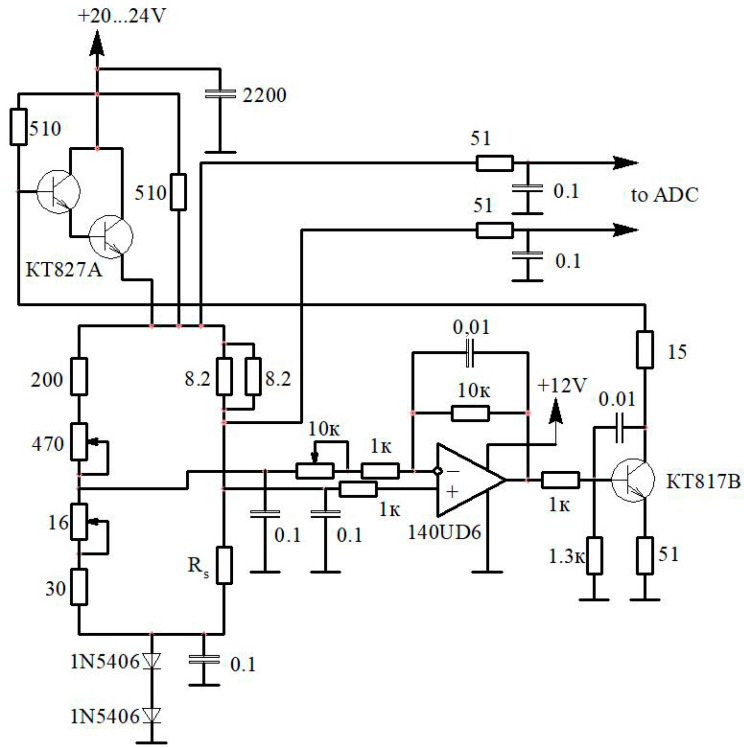
Electronic circuit of the developed constant-temperature hot-wire anemometer: ADC–analog-to-digital converter; KT827A–transistor; 1N5406–transistor; 140UD6–voltage amplifier; KT817B–transistor; *Rs*–resistance of the hot-wire anemometer sensor.

To study the gas-dynamic characteristics of pulsating gas flows with a high degree of unsteadiness, it was decided to develop a constant-temperature hot-wire anemometer circuit with Russian components (Figure 2). However, it should be noted that the scheme does not contain any complex (innovative) components. Therefore, a measuring device based on this circuit can be easily assembled from other similar components from any manufacturer.

To operate the hot-wire anemometer, a stabilised power supply unit with a voltage of 20–24 V and a current of 1.5 A was used. The hot-wire anemometer’s output signal was an analogue signal up to 5 V. This signal was fed into an analogue-to-digital converter from National Instruments and further processed on a personal computer with custom-made software (LGraph2 v. 2.35.20).

**Figure 3:**
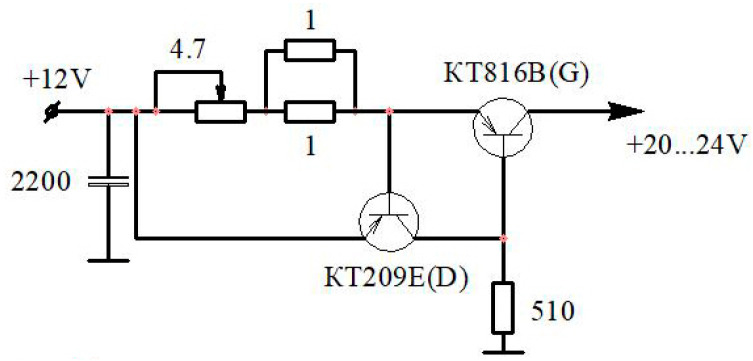
Electronic circuit of the filament protection unit (the sensitive element of the hot-wire anemometer sensor) in setup mode: KT816B(G)—transistor; KT209E(D)—transistor.

For the convenience of setting the hot-wire anemometer before work (setting the initial strength of the current on the sensitive element of the sensor), a special unit was introduced into the electronic circuit to protect the filament from overheating (Figure 3). The protection unit was needed due to the fact that there are cases when, while setting the initial current level, short-term surges occur on the sensitive element (filament), which lead to the sensor’s failure (the burnout of the filament). This unit was designed to protect the sensitive element by limiting the filament heating current when setting up the hot-wire anemometer before operation. This is especially important when studying flows with high pulsation amplitudes since the initial current strength is set at zero gas velocity.

The filament protection unit was also assembled with Russian components. Several key features of the protection unit should be noted: (1) simple and reliable electrical circuitry; (2) common and cheap components; and (3) the avoidance of accidental overheating (breakage) of the expensive hot-wire anemometer sensor. Thus, it is possible to increase the reliability of the thermal anemometry method and reduce financial costs for the purchase and repair of sensors through the use of this unit. A patent from the Russian Federation has been received for the electronic circuit of the hot-wire anemometer with a protection unit (patent No. 81338 RU).

**Figure 4:**
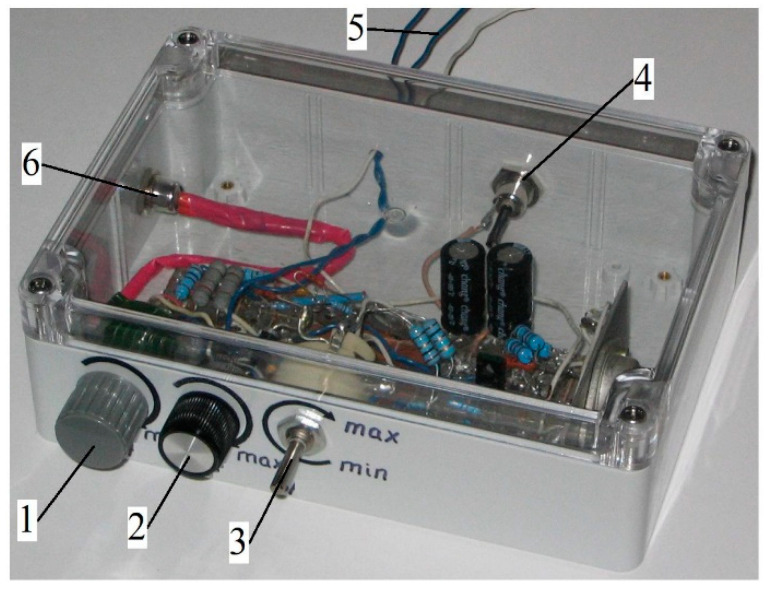
Photograph of the developed constant-temperature hot-wire anemometer: 1—fine adjustment of the initial current strength on the sensor; 2—rough adjustment of the initial current strength on the sensor; 3—limitation of the initial current for the hot-wire anemometer sensor (protecting the filament from overheating); 4—connector for connecting the power supply; 5—output to an analogue-to-digital converter; and 6—connector for connecting the hot-wire anemometer sensor.

The appearance of the developed hot-wire anemometer is shown in Figure 4.

**Figure 5:**
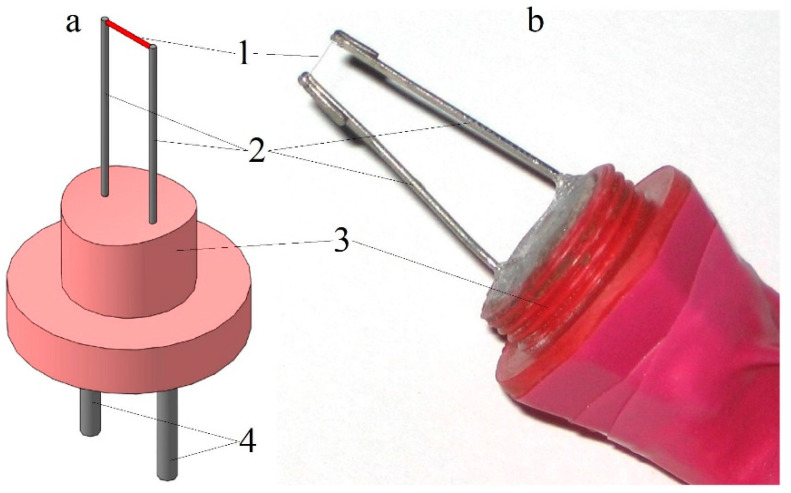
Three-dimensional model (**a**) and photograph (**b**) of a hot-wire anemometer sensor for measuring instantaneous values of the airflow velocity in gas-dynamic systems: 1—sensitive element of the sensor (nichrome thread); 2—current-conducting holders; 3—sensor base; and 4—output terminals.

The design of a hot-wire anemometer sensor for measuring instantaneous gas flow velocity values is shown in Figure 5.

A nichrome thread with a diameter of 5 μm and a length of 5 mm was used as a sensitive element. Current-conducting rods with a diameter of 1.0 mm and a length of 5 to 50 mm were used as filament holders. They were placed in a sleeve and filled with epoxy glue. The filament was spot-welded to the rods. Wires were soldered to the protruding ends of the rods, which were connected to the input of the hot-wire anemometer. The resistance of the hot-wire anemometer sensor in a cold state was 2.0–2.5 Ω.

Thus, an electronic circuit for a hot-wire anemometer operating according to the constant-temperature method was developed, with a unit for protecting the sensor’s sensitive element from overheating during pre-operation setup. This measuring system was designed to determine the instantaneous values of gas flow velocity in gas-dynamic systems with complex configurations. The design of a hot-wire anemometer sensor for the developed hot-wire anemometer was also proposed.

## 3. Evaluation and Description of the Technical Specifications of a Constant Temperature Hot-Wire Anemometer

Before using the developed hot-wire anemometer, it was tested, and its technical specifications were checked.

**Figure 6:**
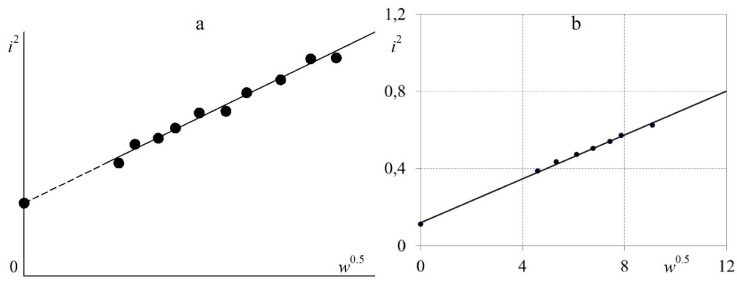
Linear dependence of *i*^2^ on *w*^0.5^ for the filament (sensitive element) of a constant-temperature hot-wire anemometer sensor (*i*—electric current strength, A; and *w*—airflow speed, m/s): (**a**) data from a monograph [1]; and (**b**) author’s data.

From the theory of the operation of constant-temperature hot-wire anemometers, it is known that with the constant electrical resistance of the filament (that is, at a constant temperature of the filament), the dependence between *i*^2^ and *w*^0.5^ should be linear [1,36]. Accordingly, for the hot-wire anemometer being developed, this linear dependence was satisfactorily confirmed via experiments during the static calibration of the hot-wire anemometer with a nichrome filament with a diameter of 5 μm and a length of 5 mm (Figure 6).

Figure 6 shows that the dependence of *i*^2^ on *w*^0.5^ for the hot-wire anemometer sensor is linear with a standard deviation of less than 0.5%.

To determine the technical specifications of the hot-wire anemometer, static and dynamic calibration was carried out. The main purpose of static calibration was to determine the dependence of the hot-wire anemometer output voltage *U* on the airflow velocity *w* in the pipe. To do this, it was necessary to correlate the readings of the hot-wire anemometer and an alternative method for measuring airflow speed. In this case, an alternative determination of *w* was carried out with a pneumatic probe based on dynamic pressure (the pressure difference). Therefore, during static calibration, the following physical quantities were measured: air temperature *T* (K), atmospheric pressure *p* (Pa), and dynamic pressure Δ*p* (Pa).

The air density under these conditions was calculated with the following formula [37]:

(1) $$\rho =\frac{p}{RT},$$

where *R* denotes the gas constant for air equal to 286.7, J/(kg K), *p* indicates the atmospheric pressure, Pa; and *T* represents the air temperature in the channel, K.

The airspeed under these conditions was determined using the following formula [37]:

(2) $$w=\sqrt{\frac{2\Delta p}{\rho }}.$$

**Figure 7:**
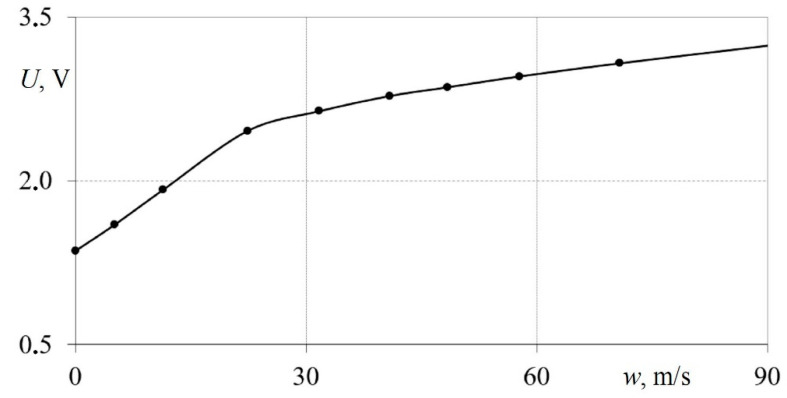
Dependence of the hot-wire anemometer output voltage *U* on the airflow velocity in the pipeline *w* (calibration curve of the hot-wire anemometer sensor).

Thus, the airspeed was measured in 10–12 modes; in parallel, measurements were obtained with the developed hot-wire anemometer. As a result, calibration curves were calculated in the form of the dependence of the voltage at the output of the hot-wire anemometer *U* on the airspeed *w* (Figure 7). For all sensors being developed, similar measurements (calibrations) were carried out two to three times to confirm the results’ reproducibility.

From Figure 7, it can be seen that the calibration curve has two linear sections. The first section is in the speed range from 0 to 27 m/s, while the second is from 27 to 100 m/s. Consequently, the developed measuring system is convenient for studying gas-dynamic processes at both low and high speeds.

To evaluate the performance of the measuring system based on the developed hot-wire anemometer and sensor, dynamic calibration was carried out. It is described in detail in [38]. The principal task of dynamic calibration was to determine the time constant of all components and the measuring system as a whole.

The time constant of the electronic circuit of a hot-wire anemometer when operating according to the constant-temperature method is significantly less than that of the direct current method [1,36]. At the same time, the frequency range of a constant-temperature hot-wire anemometer is up to 50 kHz, which fully covers gas-dynamic processes in most industrial machines and heat engines. These technical specifications provide the constant-temperature method with a significant advantage when it comes to obtaining measurements of flows with a high relative turbulence intensity and a high degree of gas-dynamic unsteadiness.

**Figure 8:**
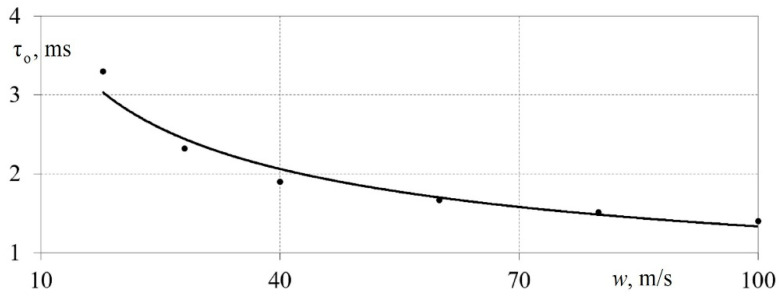
Dependence of the time constant τ_o_ of a measuring system based on a hot-wire anemometer and a filament sensor on the airflow speed *w* [38].

Figure 8 shows the dependence of the hot-wire anemometer time constant τ_o_ on the airflow velocity *w* for the developed measuring system (a hot-wire anemometer with a sensor). From Figure 8, it can be seen that the time constant τ_o_ of the measuring system ranged from 1.3 to 3.3 ms. In this case, τ_o_ decreased with an increased airflow speed [38]. This pattern was confirmed using classical data from other authors [1,36].

The key technical parameter of the measurement system is the relative uncertainty of the experiment. The assessment of the relative uncertainty of the measured quantities for this study is depicted in Table 1. The assessment of the uncertainty of physical quantities was carried out between the root-mean-square errors of the original and calculated quantities in accordance with the methods of [39].

Thus, according to the results of the static and dynamic calibration of the measuring system based on a hot-wire anemometer and sensor, the following key technical specifications were determined:

− The correct functioning of the measuring system was confirmed by comparing individual indicators with other authors’ data;
− A calibration curve for the developed measuring system was obtained;
− The time constant of the measuring system was determined;
− The relative uncertainty of the experiment was calculated to study gas flows in a pipe.

## 4. Solving Applied Problems (the Measurement of Pulsating Gas Flows)

**Figure 9:**
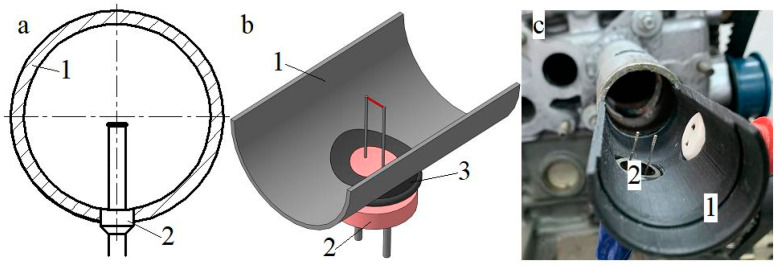
Diagram (**a**), three-dimensional model (**b**), and photograph (**c**) of installing a hot-wire anemometer sensor for measuring airflow velocity into a pipeline: 1—pipe; 2—hot-wire anemometer sensor; and 3—auxiliary spacer.

The developed measuring system was successfully used to obtain local instantaneous velocity values *w_x_* for both stationary and pulsating airflows in various gas-dynamic systems. The method for installing a hot-wire anemometer sensor in a pipeline to measure the speed *w_x_* is shown in Figure 9.

**Figure 10:**
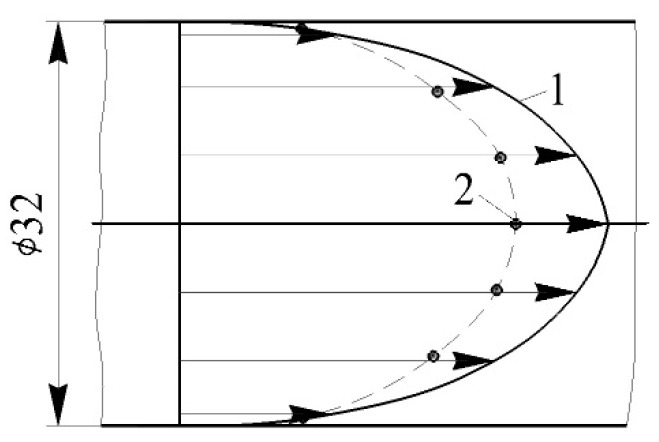
Comparison of velocity profiles in a long, straight pipe (inner diameter: 32 mm) at a distance of 50 calibres: 1—experimental data from [40]; 2—the author’s experimental data based on the proposed measuring system.

A comparison of stationary airflow velocity profiles in a long, round pipe is shown in Figure 10. Data from the classic book by I.E. Idelchik [40] were taken as a basis for comparison.

It was established that the type of velocity profiles coincided within acceptable limits (Figure 10). Quantitative differences did not exceed 5.5%. This difference may have been due to the fact that, in the author’s experimental studies, there was a different way of entering the airflow into the pipeline. Accordingly, the proposed measuring system reliably records changes in airflow velocity along the entire pipeline profile.

**Figure 11:**
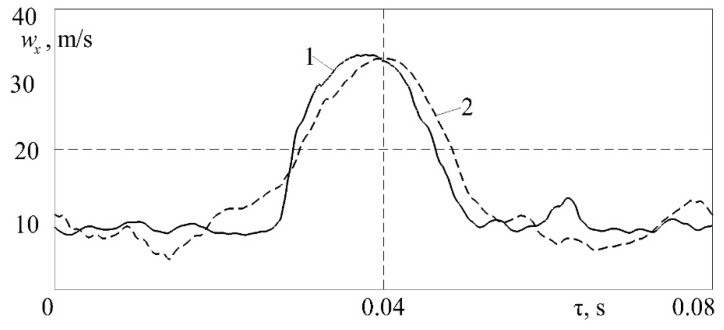
Dependences of the airflow velocity *w_x_* on the time τ in the intake system of the piston engine (piston diameter—210 mm; piston stroke—210 mm; and crankshaft speed—1500 rpm): 1—the author’s experimental data based on the proposed measuring system; 2—experimental data from [41].

A comparison of the unsteady airflow velocity in the intake system of a diesel engine for one duty cycle is shown in Figure 11. The basic dependence *w_x_* = *f* (τ) was obtained from a classic monograph by B.Kh. Draganov [41].

Figure 11 shows that the general form of the regularity *w_x_* = *f* (τ) coincided. Quantitative differences in the values of the airflow velocity did not exceed 7.0%. Thus, the proposed measuring system also reliably records pulsating airflows.

**Figure 12:**
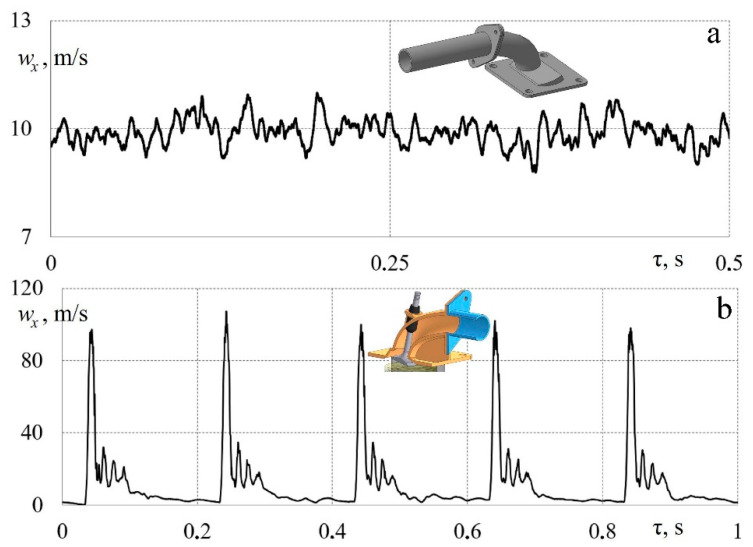
Dependences of changes in local airflow velocity *w_x_* in a piston engine’s exhaust system over time τ: (**a**) stationary flow with an average speed *w* = 9.9 m/s (a curvilinear pipeline with a fixed valve); (**b**) pulsating flow (a curvilinear pipeline with a poppet valve operating at a frequency of 5 Hz).

Figure 12 shows an example of measuring instantaneous values of airflow velocity in a piston engine’s exhaust system in both stationary and pulsating driving modes.

Figure 12a shows the dependence *w_x_* = *f* (τ) for airflow through the exhaust system with a fixed valve position in a fully open state, that is, a stationary purge of the system. In this case, the flow turbulence intensity was 4.5%, which corresponds to the average values for pipelines. Figure 10b shows the dependence *w_x_* = *f* (τ) for the unsteady case when the poppet valve in the exhaust system opened and closed with a frequency of 5 Hz (crankshaft speed: *n* = 600 rpm). More detailed data (different modes and frequencies) can be found in [42]. The data obtained confirm that the measuring system based on a hot-wire anemometer and sensor reliably processes non-stationary gas-dynamic processes in a piston engine’s exhaust pipeline. The data in Figure 12b are well in line with the results presented in previous publications [24,41].

**Figure 13:**
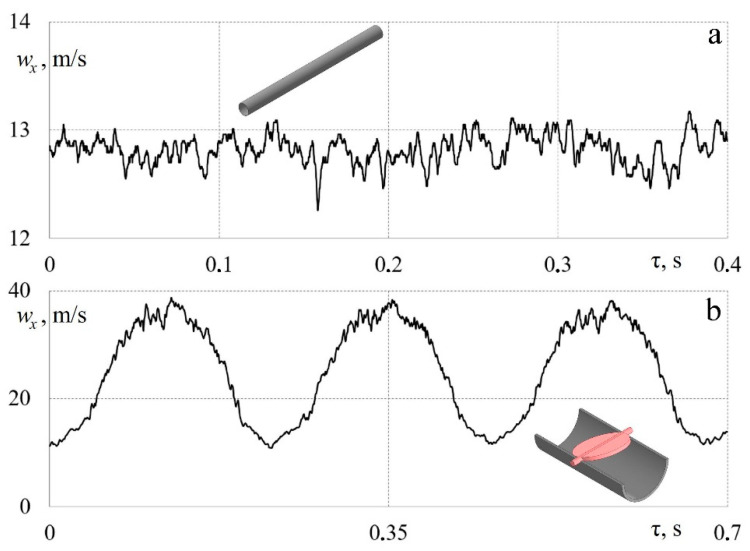
Dependences of changes in local airflow velocity *w_x_* in a long pipeline over time τ: (**a**) stationary flow with an average speed of *w* = 12.7 m/s (a straight pipeline); (**b**) pulsating flow with a frequency of 4.7 Hz (a straight pipeline with a rotating damper).

Figure 13 shows the patterns of changes in airflow velocity in a long and straight pipe in both stationary and non-stationary modes. In this case, flow pulsations were created by rotating the valve at the initial section of the pipeline.

Figure 13a shows the dependence *w_x_* = *f* (τ) for airflow in a long and straight pipe in a stationary mode. In this case, the flow turbulence intensity was ~3.5%, which corresponds to the average values for pipelines. Accordingly, the hot-wire anemometer correctly records the instantaneous flow velocity values.

Figure 13b shows the dependence *w_x_* = *f* (τ) for a straight pipeline with a rotating damper inside with a frequency of 4.7 Hz. The data obtained confirm that the measuring system correctly records large amplitudes of speed changes from 10 to 40 m/s and small fluctuations in *w_x_* within 0.5 m/s or less. This indicates that the hot-wire anemometer can be used for a detailed study of gas-dynamic effects with different scales and intensities of flow turbulence.

**Figure 14:**
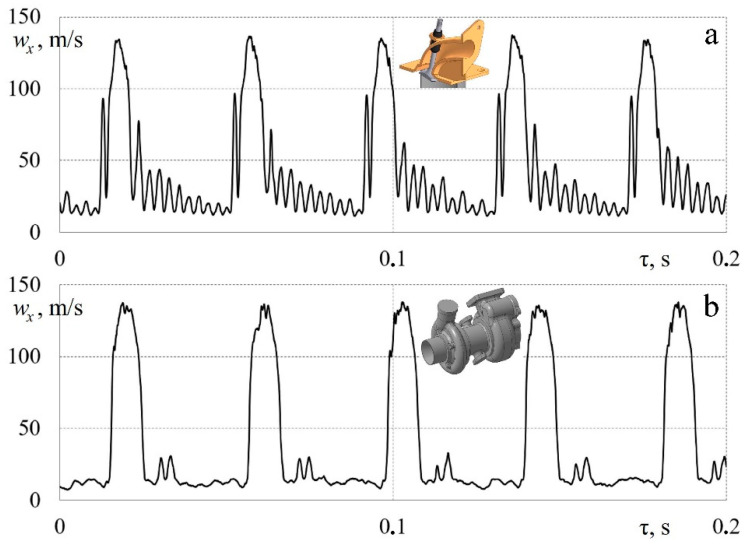
Dependences of changes in local airflow velocity *w_x_* in a piston engine’s intake system in time τ: (**a**) pulsating flow (a curvilinear pipeline with an operating poppet valve with a frequency of 25 Hz without any mechanical impact on the flow); (**b**) pulsating flow with an external influence (a curvilinear pipeline with a poppet valve operating at a frequency of 25 Hz with the action of turbocharger blades on the flow at a rotation speed of 46,000 rpm).

Figure 14 shows the patterns of changes in airflow speed in the intake system in a pulsating mode (the operation of a poppet valve) with an additional external influence from a turbocharger’s compressor blades.

Figure 14a shows the dependence *w_x_* = *f* (τ) for a pulsating flow when a poppet valve operates in the intake system with a frequency of 25 Hz (crankshaft speed: n = 3000 rpm). The damped oscillations after the main peak are reverse-pressure waves that occur in the intake manifold after abrupt valve closure [43,44]. The measuring system qualitatively records all speed variations for a given case.

Figure 14b shows the dependence *w_x_* = *f* (τ) for a pulsating flow with a frequency of 25 Hz and the simultaneous external (mechanical) action of the turbocharger blades (more data can be found in article [45]). The turbocharger rotor rotated at a frequency of 46,000 rpm, while the compressor had 12 blades. Consequently, the measuring system recorded small fluctuations in speed in the area of maximums. However, most of the small air fluctuations went unnoticed because their frequency exceeded the time constant of the hot-wire anemometer and sensor.

Thus, three applied examples of using the developed measuring system for different physical mechanisms of the creation of gas-dynamic unsteadiness in gas flows have been shown. It has been shown that the hot-wire anemometer with sensors correctly measures both large pulsations of airflow velocity and small fluctuations within the measuring system’s time constant.

## 5. Conclusions

Based on the presented data, the following key results could be formulated:

− An electronic circuit for a constant-temperature hot-wire anemometer with a unit for protecting the sensor filament from overheating during pre-operation setup was developed;
− The design of a hot-wire anemometer sensor was proposed for measuring instantaneous values of gas flow velocity in pipeline gas-dynamic systems;
− Based on static calibration, the correct operation of the hot-wire anemometer with a sensor was confirmed by comparing tests with data from other authors;
− The dependence of the airflow speed on the output voltage of the hot-wire anemometer for the developed measuring system was identified;
− Based on dynamic calibration, the time constant of the measuring system was determined;
− Based on applied studies of both stationary and pulsating airflows for various gas-dynamic systems, it was shown that the developed hot-wire anemometer with sensors correctly measures both large velocity pulsations and small fluctuations within the time constant of the measuring system.

Thus, the scientific novelty of this work lies in three key aspects:

(1) A scheme for a constant-temperature hot-wire anemometer with an original block for protecting the filament (the sensitive element of the hot-wire anemometer) from overheating before starting work was proposed (this increases the reliability of the thermal anemometer method);
(2) An algorithm for determining the main technical characteristics of a constant-temperature hot-wire anemometer and testing its performance was shown;
(3) A set of fundamental and applied problems in the field of gas dynamics was presented, which can be studied using the thermal anemometry method (this helps to expand the knowledge base about the gas-dynamic characteristics of flows in systems of complex configuration).

## Figures and Tables

**Table 1:** Relative uncertainty of physical quantities.

Parameter	Instrument	Relative Uncertainty, %
Barometric pressure	Barometer	0.1
Pressure drop in flow	Micromanometer and transducer	2.5 ^a^
Air temperature	Thermocouple and potentiometer	1.0
Airflow speed in the channel	Constant-temperature hot-wire anemometer	5.1 ^b^
Thermophysical properties of substances	Thermophysical reference book	2.0

Note: ^a^ the uncertainty is given, taking into account the calibration error (1.1%) and conversion to digital code (2.2%); ^b^ the uncertainty is given, taking into account the error in converting the analogue signal to a digital code.

## Data Availability

Data are contained within the article.

## Nomenclature

ADC	analogue-to-digital converter
H-WA	hot-wire anemometer
ρ	air density, kg/m^3^
*R*	gas constant for air, J/(kg K)
*p*	atmospheric pressure, Pa
*T*	air temperature, K
Δ*p*	dynamic pressure, Pa
*w_x_*	local air velocity, m/s
*w*	average airflow velocity, m/s
*n*	crankshaft rotation frequency, rpm
*U*	electrical voltage, V
*i*	electric current, A
τ*_o_*	time constant, s
τ	time, s
